# Using Long-term Capture Data to Predict *Trogoderma variabile* Ballion and *Plodia interpunctella* (Hübner) Population Patterns

**DOI:** 10.3390/insects10040093

**Published:** 2019-03-30

**Authors:** Alison R. Gerken, James F. Campbell

**Affiliations:** USDA, Agricultural Research Service, Center for Grain and Animal Health Research, 1515 College Ave, Manhattan, KS 66502, USA; james.campbell@ars.usda.gov

**Keywords:** warehouse beetle, Indianmeal moth, stored product pests, integrated pest management

## Abstract

Insects can infest facilities that house and process post-harvest grains and grain-based products. Integrated pest management tactics rely on tracking insect populations and using this information to select and target management tactics. Our ability to predict when and where to best focus treatment relies on an understanding of long-term trends, but often any available monitoring data are limited in its duration. Here we present data collected over a 10-year period at a flour mill in the central part of the United States. Using traps placed both inside and outside a flour mill and baited with pheromone-lures for *Plodia interpunctella* (Hübner), Indianmeal moth, and *Trogoderma variabile* Ballion, warehouse beetle, we examine environmental and spatial variability in insect captures. We find that both species, inside and outside the mill, are highly influenced by seasonal patterns, with peaks of insect captures during the warm season (April through September). There is also consistency across time and space in trap capture for *P. interpunctella* with traps in an open location consistently capturing high numbers of insects. In contrast, *T. variabile* lacked consistency in trap capture but were most often not found in the same trap locations as *P. interpunctella*. Fumigations conducted within the facility appeared to have little impact on insect captures inside, with dynamics appearing to be driven more by broader seasonal patterns in activity. These data and analyses suggest that there is a larger population of these insects that are readily moving in and out of the structures, while fumigation treatments are only impacting a small portion of the overall population and tactics targeting immigration may be an important addition to the pest management program.

## 1. Introduction

A range of insect pests are found in buildings where grain and grain-based products are processed and stored, such as flour mills, processing facilities, warehouses, and retail stores and infestations can cause large monetary costs each year in lost product [1]. The presence of insects inside these structures can lead to infestation of grain and finished products which can contaminate the food or lead to infestation of food material that accumulates in the building structure creating unsanitary conditions that can lead to later product infestation. Managing these pests can be a challenge as these facilities are spatially complex, dynamic, and features can vary considerably among locations. A foundation of an effective pest management program is a good monitoring program. Information from monitoring programs can be used to evaluate effectiveness of prevention programs that include tactics such as sanitation and structural modification and determine when additional tactics such as fumigation may be needed [2,3,4,5,6,7]. Monitoring programs can also help with determining the source of insects and help with targeted responses. For example, insect migration can play a substantial role in recolonization following fumigation or sanitization [6,7,8,9]. To better assess the potential effects of pest management techniques, monitoring can be used to quantify areas where populations of these insect pests are increasing in number and may warrant further attention or treatment [6,10].

Each food facility location can have distinct spatial features, but it is important to find common approaches and general conclusions. One such approach is that at all facilities it is helpful to assess insect activity both indoors and outdoors, since patterns in insect captures helps us understand where there are significant insect reservoirs and potential for migrant insects to recolonize indoor areas following treatment. For example, at one food facility insect captures inside and outside of the building were correlated for *Trogoderma variabile* Ballion, but not for *Plodia interpunctella* (Hübner), and more *T. variabile* were captured overall than *P. interpunctella* [9], but at another location, indoor and outdoor captures of *P. interpunctella* were correlated [11,12]. Some patterns that have been found to be consistent at several different facilities include less insects outside of food patches, greater numbers on the outside or edges of food bulk, and higher captures near doors, windows and loading docks when outside activity is high [8,13,14].

There have been many monitoring studies in food facilities and several have investigated factors such as the number of traps needed for monitoring insect pests as well as the economic cost to employing these traps, but they have tended to focus on only a few years of data or monitoring only part of a facility [9,11,15,16]. Together, these studies suggest a large amount of variation in insect capture among different species [14] and locations [8,15,17], highlighting the importance of targeted integrated pest management (IPM) plans based on specific conditions at a facility [9,10,18,19]. These studies often do not find good predictors of past trends based on factors such as temperature or consistent patterns of trap captures based on location, but these predictors may be stronger with longer-term datasets. Studies that evaluate pest population patterns over multiple years and under different environmental conditions may provide a more complete picture of the factors that contribute to the spatial and environmental differences in insect captures [20]. In addition, multi-year comparisons will provide us information on the consistency of insect spatial distribution and aid in the development of IPM plans [21,22].

Spatial patterns of insects can be greatly influenced by landscape structure, with differences between indoors and outdoors being a major landscape feature [8,9]. Much of the long-term trapping data from stored product facilities is focused on *Tribolium castaneum* (Herbst), which tend to be found primarily inside of facilities and are not as mobile as some of the other stored product pests such as *P. interpunctella* or *T. variabile* [6,7,9,23,24]. Common features in a food facility such as doors, loading docks, and windows, can be used to connect interior and exterior sub-populations [8,10,14,25,26]. Spatial analysis and contour mapping based on insect captures and then superimposed onto floorplans allows researchers to identify patterns in distribution and correlate features of the facility with locations of high numbers of insects [10,11,20,22,27]. However, relationships between correlated environmental or landscape features at a trap location with consistently high or low insect captures are generally weak, which calls into question if these patterns can be meaningfully applied across facilities. Given the high level of short term and localized variation in captures [19], long-term monitoring data sets may provide a mechanism to develop a deeper understanding of patterns of activity and how they might be impacted by management tactics, such as fumigation or sanitation, and environmental variables. Broad seasonal patterns in activity have been reported [12,15,23,28] and environmental variables can vary between inside and outside of a food facility. Both indoor and outdoor factors can influence immigration and emigration and tracking their influence can also be a valuable source of understanding insect infestation changes [23]. However, environmental variables may not be the greatest factors influencing insect captures in part because factors such as grain movement, sanitation, or treatments have larger, quicker impacts on pest abundance than temperature or other seasonal changes [6,8].

Two stored product insect pests, *Plodia interpunctella* and *Trogoderma variabile*, are of particular interest because they are often captured in high numbers both in and around food facilities and show strong seasonal patterns in abundance. They have some important differences and provide two different life history and habitat colonization strategies in storage and milling facilities [29,30,31]. *Plodia interpunctella,* Indianmeal moth, is a strong flier and a previous study suggested that *P. interpunctella* captures inside buildings even after fumigations and colonization was mostly via infested product instead of immigration from outdoor populations [32]. However, other research has pointed to immigration through open doors and other routes of entry as a common method for infestation [8,9,14]. *Trogoderma variabile*, the warehouse beetle, has flying capabilities starting at 12–16 °C [33] and can move significant distances within and outside of a facility [9,10]. It is also caught both inside and outside of milling and storage facilities [8,9,12,34]. Both species infest processed wheat as well as other stored product commodities and can be particularly destructive pests. These species also have commercially available pheromone lures and are commonly monitored using pheromone traps to assess populations inside and outside of mills and warehouses, although most published data are on relatively short-term time scales [8,9,11,12].

Here, we analyze 10 years of monitoring data, capturing over 345,000 *P. interpunctella* and 270,000 *T. variabile* in pheromone baited traps, by comparing trends with environmental variables and analyzing spatial variability in captures. Monitoring was conducted at a wheat flour mill and with these data we assessed the relationship between insect captures and environmental variables such as temperature and precipitation. We also tracked insect capture consistency at different spatial scales and evaluated the impact of structural fumigation events on insect populations. In addition, we evaluated if there are threshold levels based on temperature or insect capture numbers that can predict risk thresholds of future population levels of insects within the flour mill.

## 2. Materials and Methods

**Figure 1:**
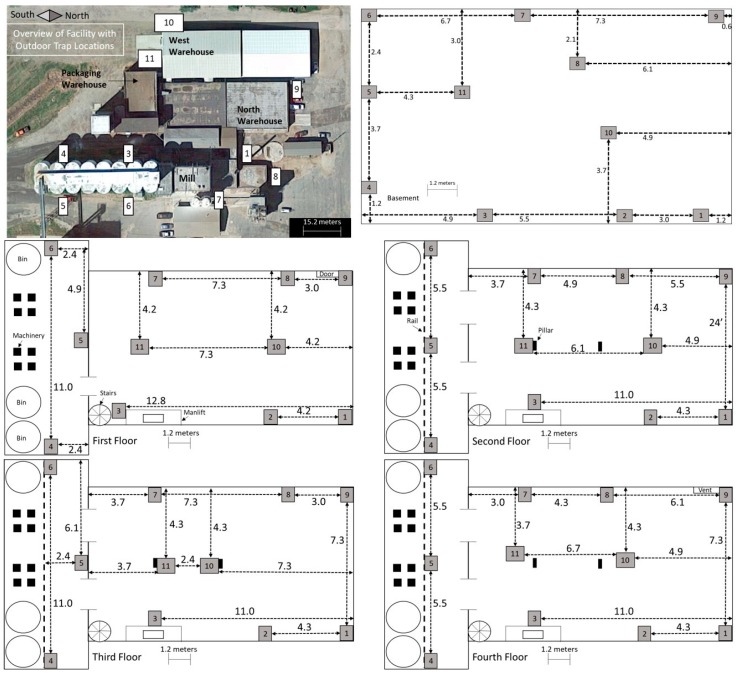
Diagrammatic layout of all outdoor and indoor trap locations for each floor. The cardinal direction key in the outdoor picture corresponds to the floor diagrams as well. Floor plans represent the basement and floors 1–4 of the mill. Machinery, bins, and pillars are indicated on the diagrams. Trap locations are indicated by numbers in white boxes (outside) and gray boxes (inside). Distances between traps and walls are indicated in meters, as is the scale key for each location. Traps in the warehouses are placed in each of the four corners and are not depicted on the diagrams.

Monitoring of *T. variabile* and *P. interpunctella* was conducted from June 2001 to February 2012 at a flour mill in central USA (Figure 1). The mill consists of five floors, including the basement floor. There is an open rectangular main area on each floor and a wider open area that spans floors 1 through 4 on the south end of the mill and contains 3 bins and 8 elevator legs. The two bins on the east are tempering bins and the west bin is for raw materials. Each floor had a similar floor plan but differed in the arrangement of structural features such as milling equipment. Each floor contains two pillars in the center of the main area and floors 1 through 4 contain stairs and a belt manlift in the southeast corner. Floor 1 has a door in the northwest corner and floor 4 has a vent to the roof in the northwest corner. Three warehouses were also monitored; they are connected to the mill with one on the north end of the facility cluster, one to the west, and a packaging warehouse to the south. Warehouses had a combination of man and overhead doors to the outside and connected to the mill.

Insects were monitored indoors using diamond traps for *P. interpunctella* and Dome traps for *T. variabile* (Trécé, Adair OK) and outside using delta traps (Scentry, Billings MT) for both species. Diamond traps and delta traps had a sticky surface to capture insects and traps were baited with *P. interpunctella* and *T. variabile* pheromones (Trécé, Adair OK). Dome traps consisted of a ramp into a deep well where insects could not exit. Diamond traps and Dome traps were placed in the same locations within the mill and warehouses. In the mill there were 11 traps per floor, with traps placed in the same locations throughout the data collection period (Figure 1). In the warehouses there were four traps total in each warehouse, each placed in a corner of the square buildings. There were 8 outdoor traps for the first three years of data collection; trap 2 was then discontinued and traps 9, 10, and 11 were added in 2004 (Figure 1). Traps were collected and replaced approximately every 14 days and the number of insects were counted; lures were replaced every 8 weeks. Outdoor traps were not deployed during winter months from December to March (2002–2003, 2003–2004, 2004–2005, and 2009–2010), December through April (2005–2006), November through March (2006–2007 and 2007–2008), and November through April (2008–2009 and 2010–2011).

Environmental data including maximum and minimum temperature, precipitation (inches), snow fall, and snow depth (inches) from the closest weather station were downloaded through the National Oceanic and Atmospheric Administration, National Centers for Environmental Information (ncdc.noaa.gov). Data were collected for every day from June 2001 to February 2012. For reporting here, degrees Fahrenheit (°F) was converted to degrees Celsius (°C). Trap capture data were used based on the date the trap was removed from inside or outside the mill, which usually standardizes to a 2-week trap capture period. To calculate the change from one monitoring period to the next, differences were calculated by subtracting captures at t from captures at t + 1, where t is the date trap was removed. Monthly or yearly data when reported or analyzed were averaged based on end date as well. To assess how our suite of variables influences insect capture we assessed the influence of our environmental factors (temperature, precipitation, and snow), location (mill floor, warehouse or outside; referred to as location for the remainder of the manuscript), season (warm or cool), and trap location (referred to as trap for the manuscript) we used stepwise models with *proc phreg* in SAS (v. 9.4, SAS Institute, Cary, NC, USA). Variables included are: year trap was deployed, precipitation, maximum temperature, minimum temperature, snow depth, location, season, and trap. We used a parameter of alpha = 0.15 for entry into the model and alpha = 0.05 for staying in the model.

Because time of year and date was influential in our model, we then assessed how season influences average insect captures. Seasons were divided as warm and cool periods based on Campbell et al. [6], where warm is April through September and cool is October through March. We examined Pearson correlations between inside and outside data first for all seasons combined followed then by year and by season. We also calculated the rates of change for each season in each year by calculating the difference in insect capture from the current trapping period to the next trapping period and calculated the percentage change. We further examined the differences in rate of change by month instead of season.

Temperature was also influential in our model, so we assessed temperature for each species to determine when the difference in insect captures began to vary from zero. To evaluate how temperature impacts the increasing risk of insect captures in a trap, we calculated the difference in insect capture from the current trapping period to the next trapping period for each temperature range using only indoor trap data for all years; we wanted to focus on identifying temperatures that indicate a consistent change was happening in trap captures so changes in trap capture that were either 0 or 100% were removed. Using *probnorm* in SAS we calculated the Z-score probability that the absolute difference from the current trapping period to the next trapping period is greater than 0 at a given minimum or maximum temperature. We then calculated the frequency of observations for each temperature for both minimum and maximum temperatures and used a cut-off of greater than 10 observations and a 0.50 probability of the difference being greater than 0 to determine our temperature of increased insect capture. We also calculated this probability based on the month.

To examine whether trap locations had consistently high or consistently low numbers of insect captures, we calculated the yearly mean insect capture for all outdoor traps, all indoor traps, and for individual traps at each location (each floor or outdoors). We counted how many years a trap was among the highest three or lowest three average counts for all indoor traps, all outdoor traps, or individual traps at each location. We then statistically compared insect captures across years with Spearman rank correlations for each trap on a given floor. We focused on year-to-year correlations and counted how many significant correlations occurred from one year to the next year for each floor and trap to examine the consistency of insect captures for each trap on a given floor. Where there is any missing data, no correlation was calculated for those year-to-year comparisons. We then used *proc nlin* to find parameters for the non-linear model comparing the average Spearman correlation for each trap to the mean insect captures for each species (Spearman correlation = θ_1_*mean insect capture/(θ_2_ + mean insect capture)). We used the Marquardt iterative method with starting parameters of θ_1_ = 0.8248280 and θ_2_ = 0 to 3.5592432 (iterated by a level of 0.2) for *P. interpunctella* and θ_1_ = 0.5450930 and θ_2_ = 0 to 0.8140484 (iterated by 0.05) for *T. variabile.* Comparisons are plotted with the default parameters of a Loess line and 0.95 confidence interval level, which uses a t-based approximation [35].

The mill was treated by fumigation a total of 15 times over the course of the 10 years of data collection by commercial applicators following labeled rates. Sulfuryl fluoride was used on June 9, 2001; June 19, 2004; and June 11, 2005. Methyl bromide was used on July 13, 2002; November 16, 2002; June 28, 2003; August 23, 2003; September 4, 2004; November 12, 2005; November 4, 2006; November 3, 2007; November 15, 2008; December 19, 2009; December 10, 2010; and December 17, 2011. Methyl bromide has been shown to be effective at all life stage in *T. variabile* [36] and *P. interpunctella* [37]. The effect of fumigations on insect captures was tracked for each trap location both within and outside the mill. For each date of fumigation, we used the two pre-fumigation trapping period counts and two post-fumigation trapping counts (usually one month each) to compare the effect of fumigation on trap catches of *T. variabile* and *P. interpunctella*. We then compared pre- and post-fumigation counts using *proc glm* in SAS with number of insects captured as the response variable and each pre- and post-fumigation group (date) and trap as main effects with trap as a random effect. Pairwise comparisons (contrasts) were used to track overall increases or decreases in insect capture between the pre- and post-fumigation periods. Percentage change from pre- to post-fumigation was also calculated to quantify the degree of change.

## 3. Results

A stepwise model was used to identify variables that potentially influence adult insect captures in traps. For *P. interpunctella*, all variables except precipitation were entered in the model (Table 1). The variables with the highest chi-square scores were location (i.e., mill floor, warehouse, or outside) (χ^2^ = 2646.11) followed by minimum temperature and year (χ^2^ = 2147.05 and χ^2^ = 794.86, respectively). For *T. variabile*, variables excluded from the model were maximum temperature, precipitation, snowfall, and corresponding trap locations on each floor within the mill (Table 1). Location was again the variable with the highest Chi-square score (χ^2^ = 2233.20) followed by minimum temperature and year (χ^2^ = 480.64 and χ^2^ = 52.50, respectively). Overall, *T. variabile* had lower Chi-square scores for placement in the models, suggesting that these variables are less reliable predictors for *T. variabile* than for *P. interpunctella*.

### 3.1. Impact of Location on Insect Counts

**Figure 2:**
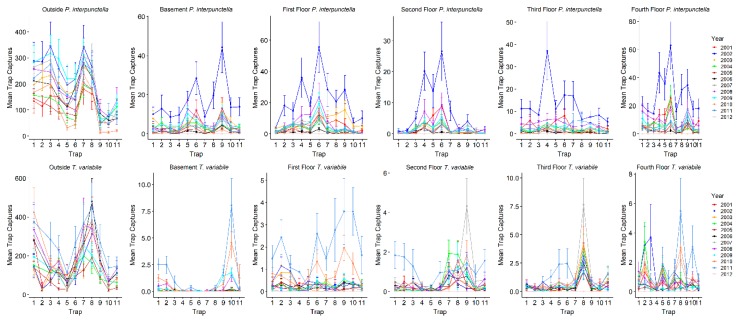
Line graphs of the mean insect captures for each species and location. *Plodia interpunctella* are in the top panels and *T. variabile* are in the bottom panels. Traps are on the x-axes and mean captures are on the y-axes; all y-axes have difference scales. Individual years are the different lines on each graph. Error bars are standard errors.

Driving the stepwise model for both *P. interpunctella* and *T. variabile* was location. The combined indoor trap (warehouse and all floors of mill) captures were highly positively correlated to outside insect captures for both species; as outside insect captures increased, indoor traps also showed an increase in insect captures. Pearson product moment correlations between inside and outside insect captures were significant for all years and seasons for *P. interpunctella* (R = 0.43–0.68, *p* < 0.0001), except 2010 (R = 0.14, *p* = 0.072), and significant for all years and seasons for *T. variabile* (R = 0.29–0.75, *p* < 0.0006). Overall, outside traps had the highest number of insects captured during both warm and cool seasons (Table 2; Figure 2). For total *P. interpunctella* caught over the total observation period, outside insect captures ranged from a total of 0 to 888 insects for a single trap with a mean of 158.3 ± 4.7 insects/trap/monitoring period (± standard error), with 2002 having the highest mean insect/trap/monitoring period captures (Table 3). In the warm season, average outside numbers were higher than in the cool season (206.9 ± 6.0 vs. 53.3 ± 4.6 insects/trap/monitoring period). Inside numbers ranged from a total of 0 to 281 insects for a single trap over the entire observation period, with an overall mean of 4.4 ± 0.1 insects/trap/monitoring period (Table 3). During the cool season the mean number captured dropped to 0.7 ± 0.04 insects/trap/monitoring period as compared to 7.9 ± 0.2 insects/trap/monitoring period caught inside during the warm season. There was significant variability in the number of insects caught in indoor traps. Warehouses had the highest mean insect captures for all indoor traps while the fourth floor had the highest mean insect captures for the floors of the mill (Table 2).

Compared to *P. interpunctella, T. variabile* had overall lower numbers of insects captured indoors but not outdoors. For all outside traps, *T. variabile* trap captures ranged from a total of 0 to 1803 for a single trap and had mean insect captures of 153.2 ± 5.7 insects/trap/monitoring period. For inside traps, total captures ranged from 0 to 48 for a single trap and averaged 0.5 ± 0.02 insects/trap/monitoring period for all inside traps. In the cool season, outside traps averaged 19.5 ± 2.8 insects/trap/monitoring period and inside traps averaged 0.07 ± 0.008 insects/trap/monitoring period, while in the warm season outside traps averaged 205.5 ± 7.4 insects/trap/monitoring period and inside traps averaged 0.9 ± 0.03 insects/trap/monitoring period. Among the inside locations, the third and fourth floors had the highest mean insect captures (Table 2).

Using the difference in insect capture from one monitoring period to the next relative to the current trap capture counts can play an important role in understanding risk levels and developing thresholds for increased monitoring or treatment [7]. Neither *P. interpunctella* nor *T. variabile* showed a consistent association between the difference in trap captures from one monitoring period to the next and mean trap capture and neither species had a mean capture threshold value associated with an increased risk of large insect capture increases. For *P. interpunctella,* for example, when trap captures were 0 insects/trap/monitoring period, trap capture differences to the next monitoring period ranged from 0 to 111 insects/trap/monitoring period (mean difference ± standard error = 0.5 ± 0.03) with the range of differences maxing out when trap counts were 54 insects/trap/monitoring period (−44 to 115 insect/trap; mean difference = 7.5 ± 20.1) (Table S1). Similarly, when *T. variabile* trap captures were 0 insects/trap/monitoring period, differences to the next monitoring period ranged from 0 to 32 (mean difference = 0.2 ± 0.009) with a maximum range when trap captures were 16 insects/trap/monitoring period (−15 to 27 insects/trap; mean = 2.3 ± 6.1).

### 3.2. Impact of Temperature on Insect Captures

Temperature was also a significant factor in the models for both *P. interpunctella* and *T. variabile*, with minimum temperature a significant factor in predicting insect capture for both species and maximum temperature significant for *P. interpunctella* only (Table 1). Both species’ insect trap capture counts had positive and significant correlations with both the maximum and minimum temperatures of the day the traps were collected. For *P. interpunctella* the correlation between insect capture and maximum temperature was always positive and ranged from 0.32–0.69 (*p* < 0.0006) and for minimum temperature ranged from 0.34–0.71 (*p* < 0.0003). For *T. variabile* correlations between insect capture and maximum temperature ranged from 0.29–0.76 (*p* < 0.002) and 0.29–0.77 (*p* < 0.002) for minimum temperature. Outside insect captures had the highest correlation to temperature for both *P. interpunctella* (R = 0.69, *p* < 0.0001 for maximum temperature and R = 0.71, *p* < 0.0001 for minimum temperature) and *T. variabile* (R = 0.76, *p* < 0.0001 for maximum temperature and R = 0.77, *p* < 0.0001 for minimum temperature). For inside locations, the *P. interpunctella* captures in the basement and first floor had the highest correlations to minimum (R = 0.52, *p* < 0.0001 for both floors) and maximum temperature (R = 0.56, *p* < 0.0001 and R = 0.54, *p* < 0.0001, respectively), while for the number of *T. variabile* captured in traps on the fourth floor and third floor had the highest correlation to minimum (R = 0.62, *p* < 0.0001 for both floors) and maximum temperatures (R = 0.63, *p* < 0.0001 for both floors).

Since temperature positively correlated with insect captures, we wanted to evaluate if there were ranges of temperatures where insect captures consistently began to change from one trapping period to the next. As temperature increased (using both the daily minimum and maximum temperatures) there was a trend for an increase in captures from one period to the next period increased and this occurred for both species. Z-scores indicated minimum temperatures where differences in insect captures from one trapping period to the next were greater than 0 were the same for both *T. variabile* and *P. interpunctella* but using maximum temperature a higher temperature needed to be reached before *T. variabile* changes were greater than 0. For *P. interpunctella*, the maximum temperature at this Z-score threshold was at 19.4 °C where there was a mean absolute difference of 5.4 ± 1.3 insects/trap/monitoring period and the minimum temperature was 6.7 °C where there was a mean absolute difference of 11.9 ± 2.2 insects/trap/monitoring period. For *T. variabile* specific maximum and minimum temperatures, Z-score probability was > 0.50 at a maximum daily temperature of 21.1 °C where there was a mean absolute difference of 6.1 ± 1.9 insects/trap/monitoring period and at a minimum temperature of 6.7 °C where there was a mean absolute difference of 3.8 ± 1.1 insects/trap/monitoring period). These results show that when these threshold temperatures are exceeded, insect captures become more variable and absolute differences from one monitoring period to the next become larger, typically between 5–11 insects/trap for *P. interpunctella* and 3–6 insects/trap for *T. variabile.*

In addition, rates of change from one monitoring period to the next that showed increases tended to cluster in the early warming months of April and May and decrease from September to October for both *P. interpuntella* and *T. variabile.* When comparing changes from one monitoring period to the next, for *P. interpunctella* April and May tended to have the greatest increases (>25% change in 5 of the 11 years of data recorded and >25% change in 9 of 11 years, respectively). In contrast, September, October, and November had the greatest decreases in captures (>−25% change in 6 out of 11 years, in all 11 years, and 6 out of 11 years, respectively). For *T. variabile* there was less consistency in monthly changes with March, April, and May having the greatest increases (>25% change in 4 out of 11 years, 4 out of 11 years, and 5 out of 11 years, respectively). September, October, and November also had the greatest declines in insect captures (>−25% change in 7 out of 11 years, 5 out of 11 years and 7 out of 11 years, respectively).

**Figure 3:**
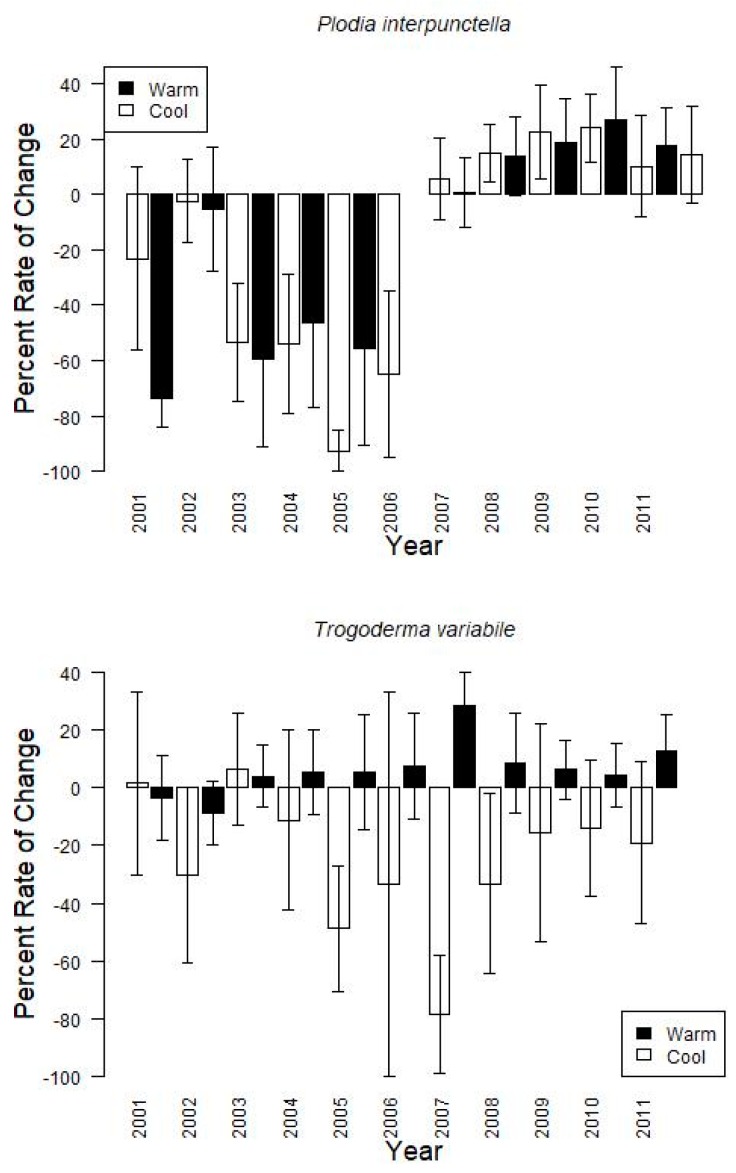
Bar graphs of the average rate of change from one trapping period to the next for each species based on indoor insect captures. The cool season is represented by white bars and warm season is the black bars. Each year is represented on the x-axis and the percent of change is on the y-axis. Error bars are standard errors.

Finally, grouping the data by warm or cool season, showed that changes from one monitoring period to the next tended to increase during the warm season (15.6 ± 15.1% on average for *P. interpunctella,* 6.5 ± 13.8% for *T. variabile*) and decrease during the cool season (−48.3 ± 23.8% for *P. interpunctella,* −25.0 ± 31.2% for *T. variabile*) with large variation among years (Figure 3). For example, during the cool seasons for *P. interpunctella,* 2002, which also tended to have the coolest temperatures during the cool season, had the greatest average decreases (−74.0 ± 10.0%) and 2003 had the lowest decrease (−2.2 ± 15.1%); 2008 had the highest increase (27.1 ± 19.4%) and 2002 had the lowest increase (0.8 ± 12.8%) in the warm season. For *T. variabile* from 2004 and onward, insect captures increased during the warm season and decreased during the cool season with 2007 having both the greatest decrease (−78.4 ± 20.4%) and increase (28.7 ± 11.6%) (Figure 3).

### 3.3. Insect Capture Spatial Consistency

**Figure 4:**
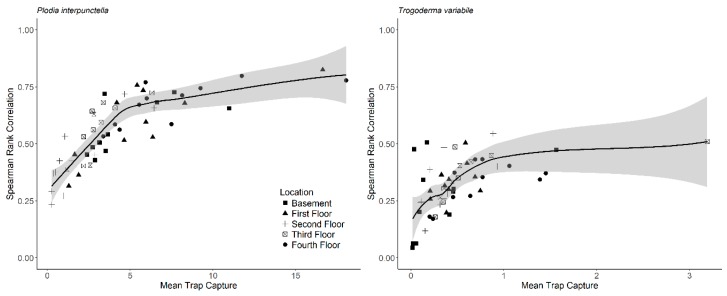
Loess line fit for mean insect capture and Spearman rank correlation for each trap and location. Spearman rank correlation is on the y-axis and represents the average correlation for each trap on a given floor from one year to the next. Mean insect capture is on the x-axis and represents the mean insect captures for each year. The line and range in gray indicate the loess line fit.

To evaluate consistency of insect captures over the years of the study, we calculated Spearman rank correlations for each location and trap for consecutive years (Table S2). We then averaged the correlations for each trap to estimate which traps had the highest consistency. Outside insect captures were the most significantly correlated for all traps for both *T. variabile* and *P. interpunctella* (Table S2). Overall, *T. variabile* had lower correlation coefficients (range: 0.38–0.44) than *P. interpunctella* (range: 0.49–0.75) suggesting that there is less consistency in insect captures for *T. variabile* than *P. interpunctella.* For *P. interpunctella* the fourth floor had the highest consistency in insect captures and traps 3, 4, 6, and 9 had high correlation coefficients between years (Supplemental Table S2). In addition, mean insect capture increased non-linearly with the correlation coefficient between years (Figure 4). The parameters for the non-linear model for *P. interpunctella* were θ_1_ = 0.78 ± 0.035 with skewness = 0.25 and θ_2_ = 1.15 ± 0.21 with skewness = 0.43 (± is approximate standard error). For *T. variabile,* parameter values were θ_1_ = 0.45 ± 0.038 with skewness = 0.46 and θ_2_ = 0.12 ± 0.044 with skewness = 0.81.

**Figure 5:**
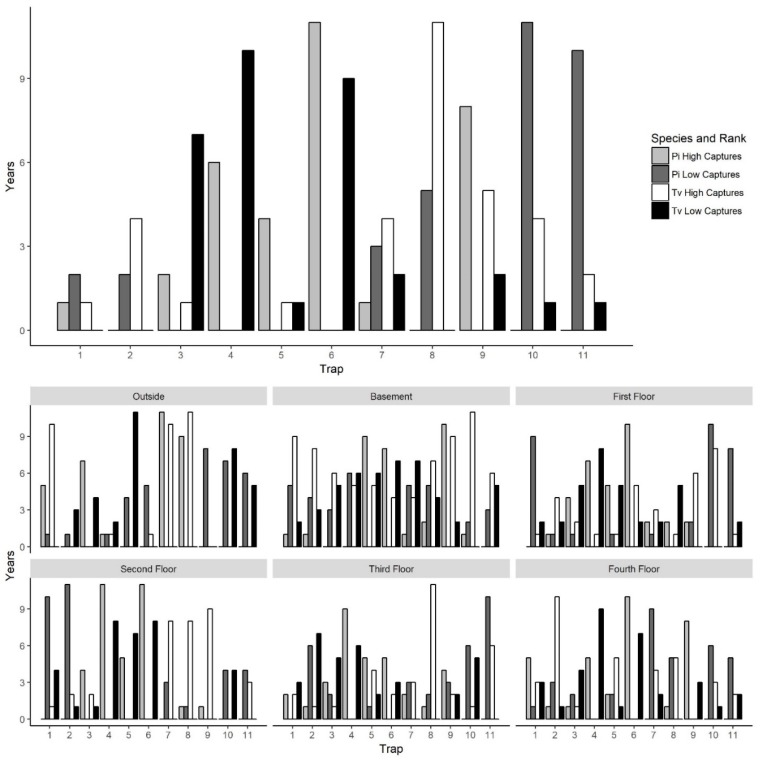
Bar graphs of number of years of trap with high and low numbers of insect captures. *Plodia interpunctella* is represented by Pi and *Trogoderma variabile* is represented by Tv. Darker colors (dark gray and black) indicate traps with low captures and lighter colors (light gray and white) indicate high captures. The number of years on the x-axis is based on how many times the trap is in the 3 traps with the highest insect captures or the 3 traps with the lowest insect captures. The top panel is all indoor traps combined and the bottom panels is each location independently.

To explore why certain traps tended to have high or low captures, we examined trap placement locations across floors since the traps were placed in the same areas on each floor and floors had similar layouts. Trap locations had significant variation in captures across years for both *P. interpunctella* and *T. variabile.* However, there were traps that typically had high or low insect captures/monitoring period. The consistency of traps across years was greater for *P. interpunctella* than for *T. variabile* and traps that had high insect captures for *P. interpunctella* did not correspond with traps with high numbers of *T. variabile* (Figure 5). For *P. interpunctella,* traps 6, 4, and 9 had the most years where they were in the top three traps with high insect captures and traps 10, 8, and 11 had the most years where they were in the bottom three in insect captures. For *T. variabile,* traps 8, 9, and 10 had the most years in the top three highest inside insect captures and traps 4, 6, and 3 had the most years in the bottom three with the lowest insect captures (Figure 5), but trap was not significant in the stepwise model for *T. variabile* (Table 1). There was little overlap between top three traps with high insect captures of *P. interpunctella* and *T. variabile* inside the mill. For example, on the first floor, traps 4 and 5 had the most years with high insect captures for *P. interpunctella* but the most years with low insect captures for *T. variabile* (Figure 5). Traps 6 and 4 may have had high *P. interpunctella* captures as they were located in an area of the mill where it was open from floors 1 to 4 and trap 9 was located by doors on the first and fourth floors, while traps 8, 10, and 11 were located out in the open, in the center of the mill floor leading to lower numbers of *P. interpunctella* captures (Figure 1).

For *P. interpunctella* and *T. variabile* traps with the highest number of captures did differ from floor to floor and patterns of high and low capture were also evident, but variable, in outside traps (Figure 5). Outside traps 7 and 8 had the greatest number of years with the highest insect captures, while traps 9 and 10 had the greatest number of years where insect captures were lowest for both *P. interpunctella* and *T. variabile*. For inside traps, trap 6 ranked in the highest three in >6 years for *P. interpunctella* for the basement, first, second, and fourth floors while traps 10 and 11 ranked in the bottom three for >5 years for the first, third, and fourth floors. For *T. variabile,* trap 8 ranked in the highest three for >5 years for the basement, second, third, and fourth floors, while traps 4 and 5 ranked in the bottom three for >5 years for the basement, first, and second floors for both traps and the third and fourth floor for trap 4 only (Figure 5).

### 3.4. Impact of Fumigation on Insect Captures

**Figure 6:**
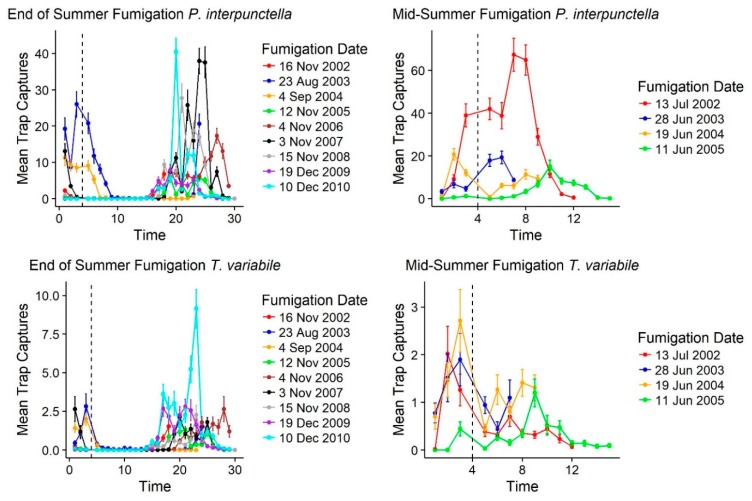
Line graphs of fumigation response. Time is represented by trapping period with fumigation date centered by vertical dashed lines at time 4 (x-axis); 3 trapping periods before, to visualize pre-fumigation counts, and 14 trapping periods after, to visualize rebound are represented. Fumigation periods are separated if they occurred towards the end of the warm season (end-of summer) or during the warm season (mid-summer) fumigations to show the differences in rebound.

Following fumigations, there was an immediate decline in insect trap captures for *T. variabile* but for *P. interpunctella* fumigations that occurred in the middle of summer there was not a sustainable drop in insect captures. For both species, fumigations at the end of summer corresponded with declines in outdoor insect captures as well. Of the 15 fumigation events that occurred over 10 years of monitoring, following fumigation there was an immediate reduction in mean insect captures for *T. variabile* (Table 4; Figure 6). For *P. interpunctella*, there were two increases in mean insect captures following fumigation; however, these fumigations occurred in the middle of the warm season (June and July) when overall numbers were highest both inside and outside of the mill (Table 2; Figure 2) and correspond to increases in outside insect captures as well for these two time points. Following end of warm season fumigations, populations tended to decline immediately (Supplemental Table S3) and only rebound at the beginning of the warm season (Figure 6). For *T. variabile*, every fumigation showed a reduction in mean number for each floor as well, except for the fourth floor on the June 11, 2005 fumigation and the west warehouse for the June 28, 2003 and June 19, 2004 fumigations (Supplemental Table S3). These increases post-fumigation are likely due to quick immigration since they are in the middle of the warm season; outside insect captures also declined at these time points as well, but outside populations were still in large numbers allowing for recolonization following fumigation. Increased captures post-fumigation occurred for *P. interpunctella* after the July 13, 2002 and June 28, 2002 fumigations for each floor except the fourth floor and the north warehouse and after the August 23, 2003 fumigation for the second, third, and fourth floors and the north warehouse (Table S3). Outside trap captures also showed an increase at these fumigation dates, suggesting a quick replacement of indoor populations of *P. interpunctella* following fumigation during these warmer months. Immediate declines in trap captures for *T. variabile* following fumigation suggest that there is more of a residential population in the mill with less immigration following treatments.

## 4. Discussion

Long-term monitoring data are often hard to obtain, but they provide an important resource for pest management and prediction of where and when insects are most likely to infest within a facility. Short-term data can provide important information to guide pest management but given the considerable variation that is often reported for food facility monitoring data it may not be as useful for making predictions about pest abundance. We see in our data, insect captures can change dramatically from year to year at each trap, location (mill floors, warehouses, or outside), and overall totals. However, by evaluating trends in captures across years, patterns may be detected that can guide how many traps may be needed, and where they should be placed, to most accurately monitor insect populations within a facility. Here we see that there are strong seasonal patterns in captures for both *P. interpunctella* and *T. variabile.* We see consistent declines in insect captures during the cool season and increases during the warm season. In April and May (*P. interpunctella*) and March, April, and May (*T. variabile*) we see insect captures increase significantly from one trapping period to the next. It is possible that the number of traps used could vary throughout the year, with more traps needed during the warm season (starting in March or April) and fewer during the cool season. This can save money and time investment in using traps as a monitoring tool in these systems.

For *P. interpunctella* and *T. variabile* we found that environmental variables, specifically temperature, have a large impact on insect captures. Both minimum and maximum daily temperature had a significant influence on insect captures for *P. interpunctella* but only minimum temperature was significant for *T. variabile,* with insect captures tending to increase as temperature increased. For *P. interpunctella* the mean difference in insect captures between sequential monitoring periods started to consistently show increases in May, with Z-scores indicating a temperature of 19.4 °C as the threshold where mean absolute differences are consistently different than zero. In contrast, *T. variabile* were slightly delayed in their threshold timing and showed high mean differences in insect capture beginning in June, and a threshold of 21.1 °C for maximum temperature when differences in captures are consistently greater than zero. Temperature has been shown to be influential in *P. interpunctella* biology with lower limits for survival and reproduction ranging from 16 to 20 °C and upper limits around 30 °C [37,38]. Temperature is also influential for *T. variabile* with warmer temperatures increasing insect captures in other studies [9] and temperatures higher than a mean temperature of 32.2 °C unfavorable to survival and a low temperature of 21.1 °C slowing down development dramatically [39].

Season also had a strong influence on the increase or decrease in insect captures from one monitoring period to the next with cool seasons tending to have negative changes in insect captures from one trapping period to the next, pointing to a downward trend in insect captures from October through March. Warm seasons tended to have positive changes and the highest percent changes typically occurred in April for both *P. interpunctella* and *T. variabile*. For monitoring purposes, these months and temperature ranges would be ideal to begin to expect insect population increases within a facility. For cooler months Z-scores indicate that differences in mean indoor *T. variabile* capture are not different from zero only in January; for *P. interpunctella*, this trend occurs from February to April. These results suggest a few patterns. First, for *T. variabile* there may be small resident populations within the facility since even during most of the cool months there are sometimes insect captures. For this species, monitoring of resident populations is important to ensure that these insects remain in check. In comparison, *P. interpunctella* captures do not change from February to April which suggests that this species is more of a migratory species into food facilities and resident populations may not need to be monitored during the cool months. Arthur et al. [11] also found a lack of *P. interpunctella* captures during cool months in a large food warehouse and that cooler temperatures had more of an effect of *P. interpunctella* than *T. variabile* [8]. Moths may also be in diapause at a larval stage, which would make monitoring them ineffective as they are not flying during this stage. However, even though air temperatures tend to drop within the mill during the cooler months, the mill is heated and is still relatively warm, maintaining the ability for resident populations to move and reproduce indoors even during cold months [6].

Location outside or within the mill and warehouses had a significant impact on insect captures for both species examined. Outside traps consistently had the highest numbers of insect capture suggesting that there are multiple sources of insects in the surrounding environment [8,9,12]. However, outside traps might also be more efficient in captures due to having more airflow increasing dispersal of the pheromone and the different trap design used. However, these differences are unlikely to generate such a large difference in captures, and other studies have also shown that these two species are found in high numbers outside of structures, which lends further to the evidence that immigration can play a large role in pest infestation within the mill [8,9,15,40]. In addition, specific outdoor trap locations tended to have the highest or lowest capture levels across years for both species (i.e., traps 7 and 8 with greatest and traps 10 and 11 with lowest captures for both species). These two groups of traps are set in different places around the outside of the mill and indicate that populations of these insects are not homogeneously distributed around the mill. Traps 7 and 8 are located near the mill itself, on the northern side of the cluster of buildings in the facility (Figure 1). Traps 10 and 11 are located on the south side of the buildings, near the west and packaging warehouses. The insect capture differences may be due to several factors, including more odor emitting from the mill as the wheat is being processed and less odor from the packaging warehouse [9]. While air flow is important in distributing pheromone plumes, differences in insect captures at these traps could indicate the importance of considering wind speed and direction relative to sources of the insect. Around this mill, strong winds are usually from the south, where traps 10 and 11 would be exposed to windy conditions possibly diminishing the effects of the pheromone lures. In contrast, traps 7 and 8 may be blocked from strong winds on the northern side of the facility and pheromone lures would be more efficient in drawing in insects. Outdoor trap 1 was also located on the north side of the facility, blocked by buildings from the south wind, and this trap also had high numbers of insect captures throughout years.

Interestingly, trap was not a significant factor for *T. variabile* as it was for *P. interpunctella*. This suggests that trap location may play a larger role for *P. interpunctella* than *T. variabile*. Indeed, trap consistency from year-to-year was lower for *T. variabile* than for *P. interpunctella* suggesting that to monitor *T. variabile* most accurately, managers may need more traps than needed to monitor populations of *P. interpunctella*. In addition, when comparing traps with highest and lowest insect captures, traps that had the highest counts for *T. variabile* did not overlap with traps with high counts for *P. interpunctella* suggesting independence in their niches or preferred habitats inside the mill. In addition, increasing mean insect capture also correlated with increasing consistency between years for both species, suggesting that traps with high captures were “hot-spots” of activity throughout the years of monitoring these species. For *P. interpunctella* this suggests that 3 or 4 traps could be enough for monitoring populations on each floor of the mill if we need to minimize the number of traps and focus our monitoring efforts.

When we combine consistency data from all traps on a floor across years, there was also variation in consistency of captures across floors. For *P. interpunctella* the upper most floor had the greatest consistency in insect captures across years. Our data indicate high mean insect captures for *P. interpunctella* on the first floor as well, so it seems as if these moths are also following the pattern of preferring the highest and lowest areas within the mill. Other behavioral analyses on *P. interpunctella* suggested that they preferred traps at both the highest and lowest levels [41]. Therefore, moth captures on the first and fourth floors of the mill might be due to a tendency to vertically orient within the mill. In addition, the top floor has openings to the outside environment, so higher activity could be associated with routes of entry, although there are considerably more routes on the lowest level relative to the top floor. The impact of the fourth-floor northwest vent, the first-floor northwest door, and the basement northwest vent door in insect capture are suggested by the higher levels of *P. interpunctella* capture at indoor trap position 9 which is near these routes of entry. This pattern also points to the influence of outdoor access to increased numbers of insects at these locations [8,13,14].

Unlike many agriculture or orchard systems there are no clear economic and action thresholds for pests within flour mills and other food facilities and with little consensus on what these thresholds might be for stored product pests [7,12]. However, there are studies that impose a threshold value of insect captures to assess the need for a response and to determine the necessity of the trap locations within an area [6,7,11]. These thresholds have worked well for resident species such as *T. castaneum* but may not work as well for more mobile species with strong patterns of movement into and out of the facility. To determine if this threshold approach would work for *P. interpunctella* or *T. variabile*, we assessed the difference in insect capture from one trapping period to the next compared to the mean insect capture and attempted to calculate the capture level when the difference started to increase as the threshold level of risk of infestation [7]. Our data did not show a consistent pattern of an increasing difference at a given threshold; even when mean captures were 0 or low, differences from the current trapping period to the next had a large range. For *T. castaneum* from this same mill, when 30% of traps captured > 2.5 beetles, trap capture was likely to begin to increase suggesting this species is a residential species and constant monitoring may be necessary to monitor risk thresholds [7]. This pattern again suggests that *P. interpunctella* and *T. variabile* are less residential species within the mill and may rely more on migration into the mill during the warm seasons.

Structural treatments such as fumigation are important for treating whole populations to inhibit the rapid recolonization potential if the treatment is unsuccessful. *Tribolium castaneum* from this facility responded strongly to fumigation treatments decreasing insect capture with insect populations rebounding around 100 days for spring fumigations and 250 days for fall fumigations [7]. Pre-fumigation levels also provide an estimate of a risk threshold level of 2.5 beetles caught per trap as mentioned above; this threshold was used to calculate risk estimates based on the change in insect capture from one trapping period to the next, suggesting that below 2.5 beetles/trap on average the chances for an increase in insect population was low (about a 0.34 insect increase) but above the 2.5 beetle capture, insect captures increased by 1.76 insects from one trapping period to the next. Together, these results suggest these populations of *T. castaneum* may be more resident than migratory with individuals following each fumigation treatment [6,7].

In contrast to the response of *T. castaneum* to fumigation treatments, although we see a decrease in *P. interpunctella* and *T. variabile* insect captures immediately following fumigation (Table 4), insect captures quickly rebound, suggesting that these insects are quickly immigrating following fumigation. In addition, insect captures indoors correspond to outdoor insect captures, even so far as to decrease at the same times that fumigations are occurring in the mill. Alternatively, insect captures soon after fumigation may also have been a failed treatment, where target concentrations and exposure times were not reached, but given the consistency of the rebound in insect captures this seems unlikely to be a major factor. Both *T. variabile* and *P. interpunctella* are susceptible to methyl bromide fumigation [36,37], but it is possible there is some resistance or repellency issues that might explain the rapid rebound. However, the high captures levels outside during treatments and the relationship between rebound and outside seasonal patterns suggests that patterns of recolonization most likely explain post-fumigation trends.

The outside or seasonal influence on insect captures is quite high and may even be more influential on interior population trends than internal environmental conditions and management tactics such as fumigation. Outside and inside insect captures also showed significant correlations and temperature was significantly correlated to insect capture, suggesting that it may be the driver of both inside and outside insect capture numbers. We do note that different traps are deployed inside and outside of the mill for these two species, but overall trends of insect captures vary consistently for both inside and outside traps. Thermal conditions have significant impacts on the reproductive capacity of insects as well as movement and flight [42,43] and so a strong influence on temperature is not surprising for these insects as well. Insects can easily migrate into the mill following fumigation, especially when doors and windows are opened for aeration [6,7]. Fumigations at the beginning of the cool season appeared to be more effective at decreasing insect captures; however, given the broader trends outside for populations to decrease this may just reflect reduced immigration. For *T. castaneum* within this facility, populations were positively correlated before and after fumigation events but there was no impact of season on foundation populations [6]. *Tribolium castaneum* in rice mills have indications of both resident populations and seasonal patterns of movement into and out of the facility influencing insect rebound following fumigations [23]. Our data show that *P. interpunctella* and *T. variabile* activity at this flour mill is driven primarily by movement into the facility with limited established and resident populations, especially for *P. interpunctella*. Understanding these dynamics is critical for selecting management tools and interpreting the results of treatments.

## 5. Conclusions

Long-term insect capture monitoring data are collected by regular monitoring programs within commercial facilities but is not commonly accessible. Analysis of long-term data can impact management decisions and focus treatment efforts within facilities like this flour mill. Assessment of trap location is also important as placing more traps than needed can be expensive and the data gathered from unnecessary traps may not contribute to the overall implications for pest management [11]. Our analyses showed that individual trap locations can be highly variable year to year, which suggests that it is important to not rely on short periods of monitoring to make decisions about where traps are needed. However, we did find locations where traps consistently captured insects, although this was stronger for *P. interpunctella* than it was for *T. variabile*. This lack of spatial consistency for *T. variabile* may provide further evidence of low-levels of resident populations within the mill. If populations of *T. variabile* are establishing throughout the mill, maintaining low levels in a variety of locations may make consistency in trap location more variable. Similarly, *T. castaneum*, which also maintain resident populations, vary in their high capture areas among years and seasons [11], but it is difficult to determine why these high infestation locations move over time, whether it is the insect moving and recolonizing, changes in food residue locations, or if it is humans moving populations within infested products [20,27]. However, more than just trends in captures needs to be considered when designing monitoring programs, since traps in critical high-risk areas can be very valuable, even if they capture few or no insects, since they can provide information on outbreaks before infestations become unmanageable. Our data also indicate that season and outside temperature may be more influential on captures of these insects inside the mill than would be expected if they were resident populations. Multi-year monitoring provides a plethora of data; distilling this down into useable information for pest management provides important clues as to the consistency and variability of insect populations within stored-product facilities.

## Figures and Tables

**Table 1:** Stepwise *proc phreg* model building of environmental, location, and timing variables for *P. interpunctella* and *T. variabile*. Possible variables entered are: season, year, location, trap, precipitation, maximum temperature, minimum temperature, and snow fall. Entry requirement *p* = 0.15; stay requirement *p* < 0.05.

***Plodia interpunctella***						
Step	Effect Entered	Effect Removed	DF	Number In	Score Chi-Square	*p*-value
1	Location		8	1	2646.11	<0.0001
2	Min. Temp		1	2	2147.05	<0.0001
3	Year		10	3	794.86	<0.0001
4	Trap		1	4	23.99	<0.0001
5	Max. Temp		1	5	23.17	<0.0001
6	Season		1	6	12.45	0.0004
7	Snow		1	7	11.19	0.0008
***Trogoderma variabile***						
Step	Effect Entered	Effect Removed	DF	Number In	Score Chi-Square	*p*-value
1	Location		8	1	2233.20	<0.0001
2	Min. Temp		1	2	480.64	<0.0001
3	Year		10	3	52.50	<0.0001
4	Season		1	4	7.89	0.0050
5	Trap		1	5	3.15	0.076
6		Trap	1	4	3.15	0.076

**Table 2:** Counts with maximum and mean for overall inside/outside numbers, inside/outside during cool season and inside/outside during warm season. Minimum values were 0 for each location and season combination. SE = Standard Error.

Species	Variable	*n*	Maximum	Mean	SE
*Plodia interpunctella*	Inside	16,505	281	4.4	0.1
	Inside, Cool Season	8052	94	0.7	0.04
	Inside, Warm Season	8453	281	7.9	0.2
	Basement	2882	137	3.9	0.2
	First Floor	2879	158	4.8	0.3
	Fourth Floor	2882	169	6.4	0.3
	Second Floor	2869	111	1.5	0.1
	Third Floor	2882	140	2.5	0.2
	North Warehouse	456	281	11.4	1.5
	Packaging Warehouse	741	217	5.5	0.7
	West Warehouse	914	256	9.3	0.8
	Outside	1723	888	158.3	0.7
	Outside, Cool Season	545	649	53.3	4.6
	Outside, Warm Season	1178	888	206.9	6.0
*Trogoderma variabile*	Inside	17,022	48	0.5	0.02
	Inside, Cool Season	8249	44	0.07	0.008
	Inside, Warm Season	8773	48	0.9	0.03
	Basement	2866	39	0.3	0.04
	First Floor	2865	34	0.5	0.03
	Fourth Floor	2869	43	0.7	0.05
	Second Floor	2856	30	0.5	0.03
	Third Floor	2865	48	0.7	0.05
	North Warehouse	984	5	0.06	0.01
	Packaging Warehouse	733	34	0.4	0.08
	West Warehouse	984	7	0.1	0.02
	Outside	1723	1803	153.2	5.7
	Outside, Cool Season	484	730	19.5	2.8
	Outside, Warm Season	1239	1803	205.5	7.4

**Table 3:** Means and maximum counts per year, inside and outside. Minimum counts in all cases is 0. SE = standard error.

Species	Variable	*n*	Maximum	Mean	SE
*P. interpunctella* Inside	2001	758	94	4.7	0.4
	2002	821	281	23.5	1.4
	2003	1664	118	5.2	0.3
	2004	1664	126	3.5	0.2
	2005	1728	110	1.9	0.2
	2006	1662	45	1.3	0.09
	2007	1600	82	3.7	0.2
	2008	1599	158	6.0	0.4
	2009	1599	131	4.3	0.3
	2010	1556	76	1.5	0.1
	2011	1598	122	3.3	0.3
*P. interpunctella* Outside	2001	117	633	145.4	17.2
	2002	110	854	254.8	23.4
	2003	167	721	203.2	15.7
	2004	190	658	111.0	10.9
	2005	220	717	104.1	9.6
	2006	160	734	147.6	13.6
	2007	170	767	170.2	15.2
	2008	140	774	183.2	17.6
	2009	140	760	213.8	18.7
	2010	159	640	100.4	12.3
	2011	150	888	171.2	17.9
*T. variabile* Inside	2001	759	11	0.3	0.03
	2002	846	24	0.5	0.06
	2003	1713	48	0.4	0.05
	2004	1712	30	0.5	0.04
	2005	1776	15	0.1	0.02
	2006	1713	17	0.3	0.03
	2007	1649	44	0.5	0.06
	2008	1649	18	0.4	0.03
	2009	1650	30	0.3	0.03
	2010	1713	32	0.7	0.06
	2011	1644	43	1.3	0.1
*T. variabile* Outside	2001	117	1698	169.8	24.3
	2002	110	1803	125.8	21.7
	2003	167	838	126.1	14.0
	2004	190	948	108.4	13.2
	2005	220	605	78.8	7.8
	2006	160	1247	174.0	18.9
	2007	170	1052	172.1	15.9
	2008	140	1708	191.2	25.4
	2009	140	947	129.5	14.2
	2010	159	1756	187.5	23.6
	2011	150	1449	263.6	28.0

**Table 4:** Change in fumigation for all inside locations combined. Before and after fumigation includes 1 month of insect capture data, or two trapping periods. SE = standard error; * = significant at *p* < 0.05.

Fumigation Date	*P. interpunctella*				*T. variabile*	
	Mean ± SE Before Fumigation	Mean ± SE After Fumigation	Percent Reduction	Mean ± SE Before Fumigation	Mean ± SE After Fumigation	Percent Reduction
13 July 2002	25.2 ± 3.3	40.4 ± 4.0	+60.31 *	1.6 ± 0.3	0.4 ± 0.081	78.10 *
16 November 2002	0.3 ± 0.09	0.00 ± 0.00	100	0.08 ± 0.03	0.02 ± 0.011	80.00
28 June 2003	5.8 ± 1.0	18.6 ± 2.1	+222.49 *	1.7 ± 0.4	0.7 ± 0.1	59.56 *
23 August 2003	17.3 ± 2.0	16.3 ± 1.8	6.08	2.0 ± 0.5	0.3 ± 0.08	86.48 *
19 June 2004	16.4 ± 1.6	3.5 ± 0.6	78.78 *	2.1 ± 0.4	0.9 ± 0.2	59.53 *
4 September 2004	8.9 ± 1.0	7.2 ± 0.9	19.41	1.6 ± 0.2	0.2 ± 0.08	90.25 *
11 June 2005	1.0 ± 0.2	0.2 ± 0.06	76.03	0.2 ± 0.08	0.2 ± 0.04	31.03
12 November 2005	0.09 ± 0.04	0.0 ± 0.0	100	0.05 ± 0.02	0.0 ± 0.0	100
4 November 2006	0.4 ± 0.1	0.0 ± 0.0	100	0.008 ± 0.008	0.0 ± 0.0	100
3 November 2007	2.0 ± 0.3	0.05 ± 0.02	97.67	0.6 ± 0.2	0.0 ± 0.0	100 *
15 November 2008	0.2 ± 0.05	0.02 ± 0.02	86.47	0.03 ± 0.02	0.0 ± 0.0	100
19 December 2009	0.0 ± 0.0	0.0 ± 0.0	0.00	0.0 ± 0.0	0.0 ± 0.0	100
10 December 2010	0.0 ± 0.0	0.0 ± 0.0	0.00	0.04 ± 0.03	0.0 ± 0.0	100
12 December 2011	0.0 ± 0.0	0.0 ± 0.0	0.00	0.04 ± 0.02	0.0 ± 0.0	100

## Supplementary Materials

The following are available online at https://www.mdpi.com/2075-4450/10/4/93/s1, Table S1: Frequency of insect count data per trap per monitoring period. Frequency is the number of traps/monitoring period that had that specific count. Mean difference is the mean difference of those traps from one trapping period to the next. The maximum and minimum differences are the maximum or minimum of the difference from one trapping period to the next. Range of differences is the minimum difference–maximum difference. The standard error is the standard error of the mean difference; Table S2: Correlations between years for all locations and traps for both *P. interpunctella* and *T. variabile*. There is a tab for each species individually and one that summarizes average correlations across years by each location; Table S3: Changes in insect trap captures before and after fumigation. Mean insect trap captures are measured 2 trapping periods before and 2 trapping periods after each fumigation event. Highlighted cells are significant changes in mean insect captures from before to after fumigation.
